# Recommendations on Periprocedural Skincare for Energy-Based Dermatologic Procedures

**DOI:** 10.1093/asjof/ojaf039

**Published:** 2025-05-09

**Authors:** Greg Goodman, Leona Yip, Cara McDonald, Frank Lin, Wenyuan Liu, John Sullivan

## Abstract

The use of energy-based device (EBD) aesthetic skin treatments is increasing in popularity. Implementation of periprocedural skincare may improve treatment-related outcomes, reduce recovery time, and reduce the frequency and severity of potential side effects. This review presents treatment recommendations to assist clinicians in optimizing integrated skincare to enhance EBD treatment outcomes. A panel of Australian 6 physicians (5 dermatologists, 1 plastic surgeon) developed 4 generalized EBD periprocedural skincare recommendations. These recommendations were divided based on 4 levels of skin barrier disruption caused by EBD treatment: none (or minimal), mild, moderate, and severe barrier disruption. The greater the disruption caused to the skin barrier, the greater care needed in choosing appropriate skincare products with suitable actives. Preprocedural skincare incorporating moisturizers, broad-spectrum sun-protection factor 50+ sunscreen, antioxidants, and niacinamide should be implemented at least 2 weeks before treatment to enhance skin barrier function and hydration. Postprocedure recommendations are made for the use of appropriate skincare actives that promote barrier repair and avoid further irritation. Prescription actives should be avoided for an appropriate period following EBD procedures; this period is dependent on the EBD treatment type and degree of skin barrier disruption it can cause. The algorithms provided here are aimed to assist clinicians in optimizing integrated skincare recommendations to enhance EBD treatment outcomes, promote healing, and reduce the likelihood of adverse events. Skincare recommendations made to patients should be based on the condition of the patient's skin, as well as the magnitude of skin barrier disruption caused by the EBD procedure.

**Level of Evidence: 5 (Therapeutic):**

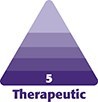

The use of energy-based device (EBD) aesthetic treatments is increasing, with the Australian skin laser market (ablative and nonablative) expected to grow by >6% by 2030.^1^ Common EBDs used for facial skin treatment include intense pulsed light, pulsed dye laser, 532 nm potassium-titanyl-phosphate, 1064 nm neodymium:yttrium-aluminum-garnet, as well as several other infrared and fractional ablative and nonablative lasers.^2^ Although considered relatively safe, these EBDs are associated with side effects such as inflamed, swollen, itchy, or painful skin, acne, microbial infection, postinflammatory hyperpigmentation (PIH), and scarring.^3^ The risk of PIH is greatest with ablative laser treatments and in darker skin types; 1 study found >90% of Asian people with Skin Phototype IV undergoing ablative fractional CO_2_ laser treatment developed mild PIH.^4^

The integration of standard skincare procedures before and after EBD treatments has been shown to reduce treatment-related side effects and improve treatment outcomes.^5,6^ To date, little guidance is available in the literature for periprocedural skincare for EBD procedures. As such, there remains inconsistency in periprocedural skincare measures implemented across centers.^2,5^ The aim of this review is to provide periprocedural recommendations and algorithms, based on the opinion of a group of experienced Australian dermatologists and plastic surgeons, to guide the use of appropriate skincare with the aim of improving treatment-related outcomes, reducing recovery time, and reducing common adverse events. Recommendations have been stratified according to the degree of skin barrier disruption caused by EBD treatment. Recommendations were developed by the authors at a face-to-face workshop on the topic.

## SKIN BARRIER SUPPORT

An intact skin barrier is essential in maintaining skin function and health. The skin barrier protects from physical, chemical, and microbial assaults and acts to prevent excess water loss.^7^ The outermost layer of the epidermis, the stratum corneum, is mainly responsible for effective skin barrier function. EBD procedures commonly cause skin barrier disruption to varying degrees. A delay in skin barrier repair can lead to persistent erythema, hyper/hypopigmentation, scarring, acne, and milia.^3^ It also increases the potential for bacterial, fungal, and viral infection, with the most common infectant being the herpes simplex virus.^8^ The risk of EBD procedure-related adverse events may be reduced through the implementation of a periprocedural skin care regimen that can reinforce and/or repair the skin barrier.^9^ The skin barrier protection effect of skincare products is dependent on factors such as the type and concentration of the active compounds used, as well as the composition of the formulation.

Immediately following skin barrier disruption, increased absorption, and dermal delivery of skin actives can occur, such as for Vitamin C and tranexamic acid. The benefits of enhanced delivery post-EBD treatment are particularly observed for low molecular weight hydrophilic or water-soluble agents. The composition or formulation of skin care products applied postlaser treatment affects both the delivery and safety.

A brief overview is given below of products recommended for use periprocedurally to provide barrier support and repair. In general, basic skin care, such as moisturizers and sunscreen, is very cost-effective and aid skin barrier healing. Active agents are usually more expensive, and their cost can vary greatly. Active agents such as retinoids, Vitamin B3, hyaluronic acids, and antioxidants are usually very affordable. Conversely, growth factors, stem-cell extracts, and peptides are premium-costed items.

### Lipids

Skin barrier permeability is regulated by the intercellular lipid-enriched matrix of the stratum corneum, which is composed of ceramides, free fatty acids, and cholesterol.^10^ As such, barrier dysfunction is partly attributed to reduced delivery of secreted lipids to the stratum corneum, leading to a decrease in the number of extracellular lamellar bilayers present.^11^ The clinical response to laser rejuvenation treatment has been attributed, in part, to differential lipid metabolism in the skin. Microarray analysis of skin biopsies from fast vs slow treatment responders revealed greater activation of lipid metabolism and keratinocyte differentiation in fast responders, who showed greater upregulation of acyltransferases, fatty acid elongases, fatty acid 2-hydroxylase, fatty acid desaturases, and specific keratins that may contribute to epidermal barrier function.^12^ Barrier restoration can be aided by the topical application of physiological lipids (eg, ceramides, fatty acids, and cholesterol), which have been shown to improve skin hydration, transepidermal water loss (TEWL), and total ceramide and cholesterol content in facial skin.^7,13^

### Moisturizers

Moisturizers form the basis of daily skincare. As well as improving the appearance of skin, some moisturizer constituents can have therapeutic effects and facilitate skin barrier repair.^14,15^ Actives found in moisturizers are categorized into 4 groups, namely emollients, humectants, occlusives, and plasticizers.^15^ Most moisturizers combine emollients, occlusives, and humectants. Emollients, such as fatty acids, fatty alcohols, cholesterol, squalene, and pseudoceramides, improve skin barrier function, membrane fluidity, and cell signaling. Humectants are low molecular weight substances, such as urea, sorbitol, panthenol, glycerol, propylene glycol, hyaluronic acid, and alpha hydroxy acids (AHAs), which increase water content in the stratum corneum. Occlusives are oils and waxes, such as mineral oil, petroleum jelly, beeswax, silicones, and zinc oxide, which form a physical layer on the skin preventing TEWL.

### Antioxidants

Some EBD skin treatments, such as photodynamic therapy (PDT), generate large amounts of reactive oxygen species. This is required for PDT therapeutic benefits, such as targeting precancerous skin cells or acne-causing bacteria. This also depletes the skin's antioxidant defenses and highlights the potential benefit of postprocedure antioxidant skin-targeted replacement to promote skin barrier repair and recovery.

Antioxidants (eg, Vitamin C, Vitamin E, L-ascorbic acid, and ferulic acid) exert several beneficial effects, including the combat of free radicals, improved skin hydration, mitochondrial protection, and cell membrane support.^16^ In a study of 150 volunteers, treatment of skin with a topical antioxidant-containing cream improved epidermal thickness, elasticity, and skin moisture.^17^ In another double-blind, placebo-controlled study of 40 volunteers, the topical administration of the antioxidants lutein and zeaxanthin reduced photodamage and lipid peroxidation, and increased the surface lipids, skin hydration, and skin elasticity.^18^ Topical antioxidants are now widely used in the field of dermatology to promote skin barrier repair and improve barrier function. There is synergistic activity in combining multiple antioxidants, such as Vitamin C, Vitamin E, and ferulic acid. It is important to select stable formulation antioxidant products because formulation instability can lead to antioxidant degradation.

### Niacinamide

Niacinamide (or nicotinamide) is a form of Vitamin B3 shown to improve stratum corneum barrier function. Niacinamide promotes the production of ceramides, free fatty acids, and cholesterol in human keratinocytes of the stratum corneum.^19^ Topical niacinamide has been shown to increase the thickness of the stratum corneum of human skin by up to 10%.^20^ Topical niacinamide has also been shown to decrease TEWL and improve dry skin, reduce hyperpigmentation, improve photoaging and wrinkles, and reduce sebum production.^19,21-25^ Niacinamide, in combination with panthenol and madecassoside, was found to ameliorate various side effects following laser skin resurfacing procedures in 3 case reports.^26^ Additionally, niacinamide, in combination with tranexamic acid and kojic acid, was shown to enhance the laser treatment of melasma.^27^

### Retinoids

Retinoids are a family of compounds derived from Vitamin A. Retinoids, such as retinol and tretinoin, have been used topically and systemically for decades for various skin conditions, particularly acne. Retinoids are thought to endow the greatest benefit when used before therapies that cause deep epidermal injury, rather than therapies that cause only superficial epidermal injury.^28^ Clinical studies have shown that the use of tretinoin before chemical peels, dermabrasion, and ablative and nonablative laser resurfacing improves subsequent procedural outcomes.^28^ Buchanan and Gilman have developed a treatment algorithm for tretinoin; preprocedural use of tretinoin has been recommended in ablative and nonablative laser facial resurfacing, surgical dermabrasion, and chemical peel resurfacing, with the tretinoin dose being dependent on the type of procedure.^28^ It is recommended that tretinoin is not started within 3 to 4 weeks before a resurfacing procedure because it can cause symptomatic dryness and exfoliation, as the facial skin has not yet had time to accommodate to its effects.^28^

### Botanicals

Although the scientific evidence for the clinical efficacy of botanicals is often scarce, they have been used to treat skin conditions for centuries and still have a place in modern medicine. Botanicals often exhibit anti-inflammatory, antimicrobial, and antioxidant properties, which are often combined in a single plant extract and may be based on several compounds rather than 1 single compound.^10,29^

Plant oils are widely used on the skin for cosmetic and medical purposes because they elicit positive physiological benefits. Plant oils can be classified into essential oils (volatile at room temperature) and fixed oils (stable at room temperature). Both essential and fixed oils have been shown to improve barrier function. Plant oils, such as jojoba, soybean, and avocado oils, remain on the skin surface, thus acting as a barrier to maintain moisture and reduce TEWL.^30^ Essential oils are thought to only penetrate the most superficial layers of the skin, forming a film to improve the epidermal water balance and improve the hydrolipidic balance and thus skin hydration.^31,32^ Care should be taken with the choice of essential oils and their concentrations because some oils can have cytotoxic properties at high concentrations.^32^ Additionally, essential oils can have a risk of skin sensitization and allergic contact dermatitis.^33^ Rather than enhance barrier function, some monounsaturated free fatty acids, such as oleic acid, may enhance skin barrier permeability and enhance chemical permeability through the skin layers.^34^

Cucumber extract is rich in vitamins, particularly Vitamins A and C.^35^ In addition to its cosmetic benefits to the skin, cucumber extract also exerts cooling, soothing, and healing effects on irritated skin.^36^

*Aloe vera* contains compounds such as salicylic acid, magnesium lactate, and gel polysaccharides and has wound-healing, anti-inflammatory, and anti-pruritic properties.^37^  *Aloe vera* can also increase skin hydration.^38^

### Sun Protection

The use of a broad-spectrum sunscreen with a sun-protection factor (SPF) of 50+ is highly advisable before and following EBD procedures. For individuals with darker skin or prone to pigmentation, a sunscreen with broad ultraviolet A (UVA) protection (including filtration of long UVA wavelengths) is advisable, with or without the addition of a tinted sunscreen or a sunscreen with iron oxide for high-energy blue light protection. Adequate sunscreen protection is recommended to reduce the chance of pigmentation disorders, such as postprocedural melasma or PIH. The incidence of PIH can be high, estimated at 30% following laser skin resurfacing in people with Skin Phototype III and 90% in people with Skin Phototype IV.^4,39^

## SKINCARE RECOMMENDATIONS FOR EBD PROCEDURES

**Figure 1.**
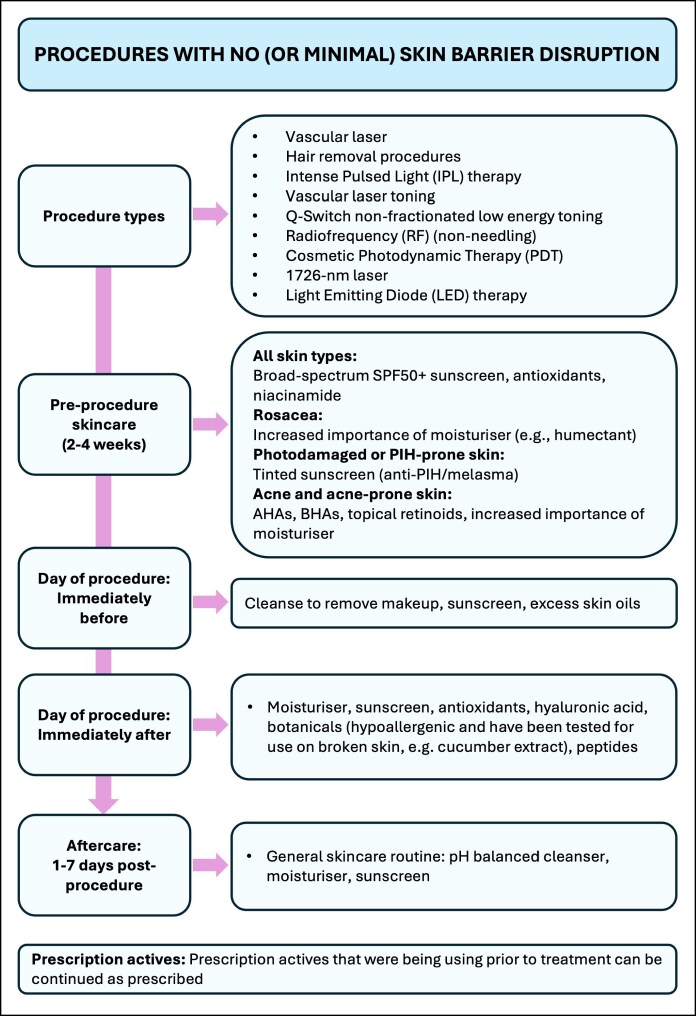
Algorithm for the integrated facial skincare for energy-based device treatments that cause no skin barrier disruption.

**Figure 2.**
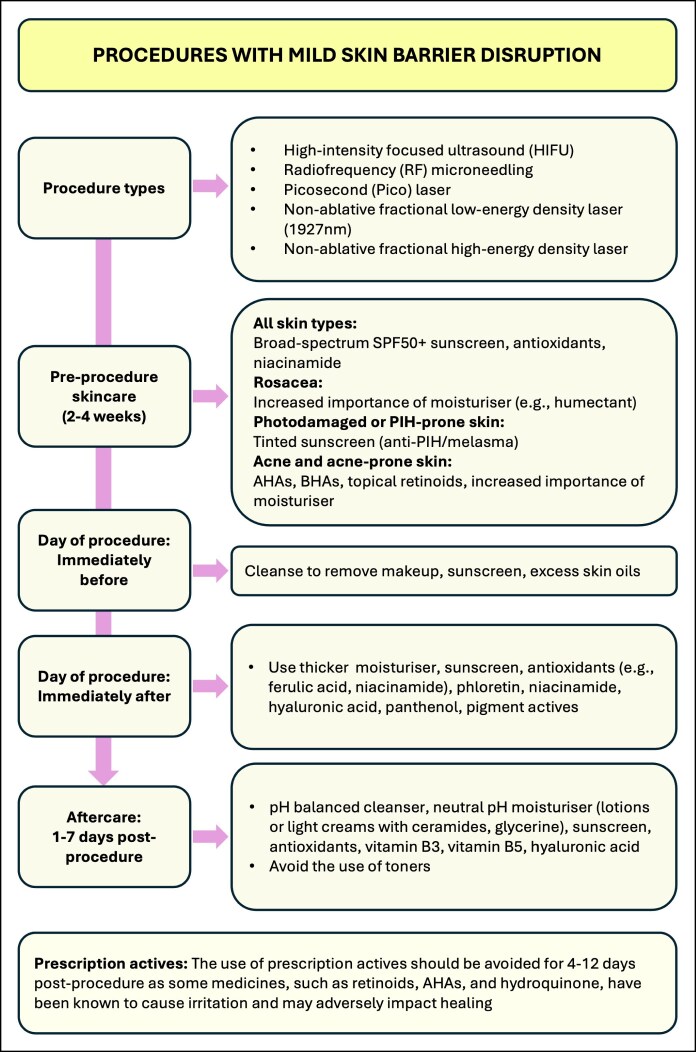
Algorithm for the integrated facial skincare for energy-based device treatments that cause mild skin barrier disruption.

**Figure 3.**
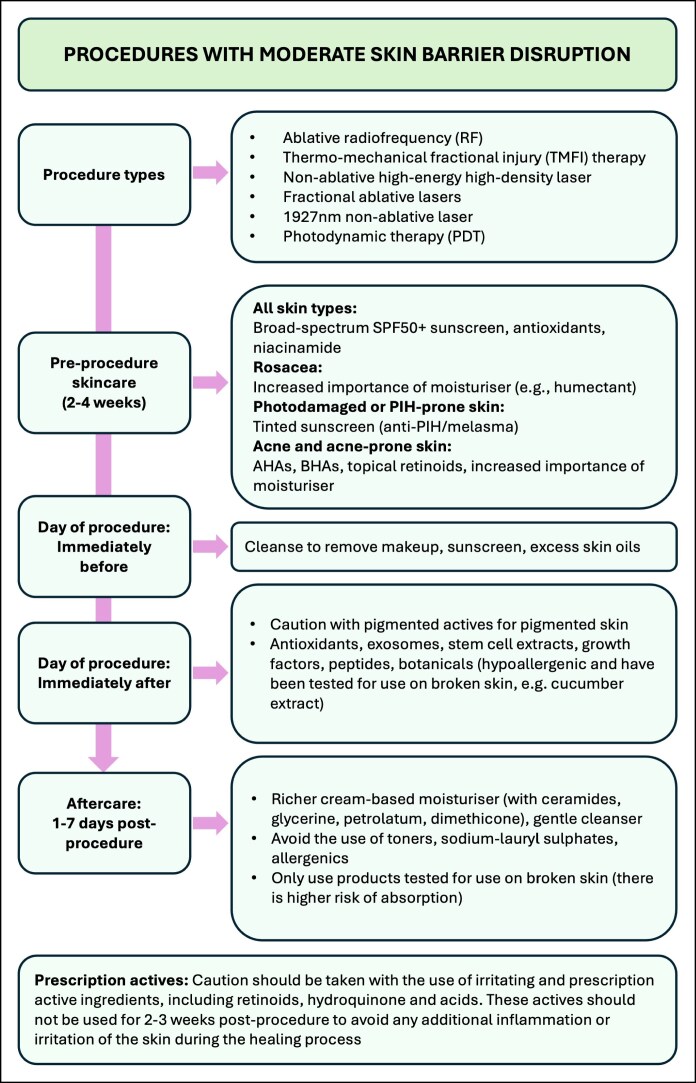
Algorithm for the integrated facial skincare for energy-based device treatments that cause moderate skin barrier disruption.

**Figure 4.**
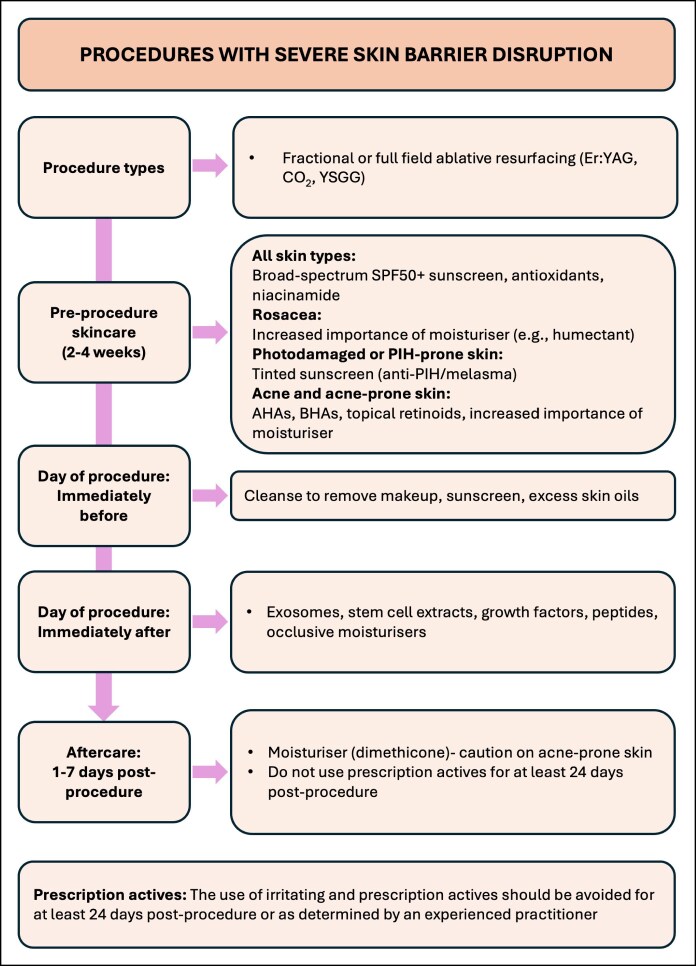
Algorithm for the integrated facial skincare for energy-based device treatments that cause severe skin barrier disruption.

Four algorithms are presented, outlining integrated skincare recommendations for energy-based skin treatments (Figures 1-4). These recommendations are based on available literature and the opinion of experienced practitioners. Recommendations are stratified into 4 categories based on the degree of skin barrier disruption caused by treatments (none [or minimal], mild, moderate, or severe barrier disruption) and are divided into preprocedural skincare and postprocedural skincare. Special consideration should be taken for inflammatory skin conditions, which should be optimally managed by a qualified practitioner before EBD treatment. EBD procedures for people with skin conditions (eg, moderate-to-severe rosacea or acne, PIH) should be performed under the supervision of a dermatologist.

### Preprocedural Skincare

The consistent application of moisturizers in the month leading up to EBD procedures is recommended for all skin types to optimize skin barrier function and hydration (Figures 1-4). The formulation choice of the moisturizer should be determined based on skin type, coexisting skin conditions, and consumer preference. Humectant moisturizers are recommended for people with rosacea and acne-prone skin to increase water content in the skin. Noncomedogenic moisturizers should be used for acne-prone skin. Moisturizers containing a combination of occlusives and humectants are advised to enhance the skin's water-retention capacity.^40^

The use of antioxidants and niacinamide is recommended for all skin types to help maintain skin barrier integrity. It is also recommended that excessive sun exposure is avoided in the month leading up to EBD procedures. Physical protective measures, such as the use of wide-brimmed hats, should be implemented. The application of a broad-spectrum SPF50 or higher sunscreen is strongly recommended to prevent sun damage to the skin. Tinted sunscreens should be recommended for melasma- or PIH-prone skin to provide protection against visible light as well as UV light. Tinted sunscreens containing iron oxide have been shown to improve hyperpigmentation.^41^ The use of hydroquinone can also be considered for people with pigmentation, PIH, or existing melasma to lighten the skin and reduce the risk of melasma recurrence or PIH.

For acne and acne-prone skin, the use of noncomedogenic products and hydrating moisturizers is recommended. Topical retinoids, AHAs, and beta hydroxy acids can be recommended to reduce inflammation, sebum production, and acne lesion formation.^42^

### Day of Procedure Skincare

#### Immediately Preprocedure

For optimal results, skin should be cleaned immediately before EBD treatment using appropriate cleansers able to remove makeup, sunscreen, and excess skin oils. An antimicrobial agent, such as chlorhexidine, isopropyl alcohol, or hypochlorous acid, is commonly used to sterilize the skin in order to reduce the chance of postprocedure infection.^2^

#### Immediately (Within 24 h) Postprocedure

Skincare requirements immediately after an EBD treatment are dependent on the type of EBD treatment used and the consequent degree of skin barrier disruption it can cause. Following EBD procedures that do not commonly cause any skin barrier disruption (Figure 1), good skincare incorporating a moisturizer, sunscreen as an absolute requirement, and antioxidants should also be advised to maximize improvements. Additionally, hyaluronic acid, peptides, and botanicals may be incorporated for appropriate patients. These agents act as additional moisturizing and messenger molecules and, in appropriate settings, may act synergistically with the EBDs used.

Following EBD procedures that cause mild skin barrier disruption (Figure 2), the use of a thicker moisturizer, sunscreen, antioxidants (eg, ferulic acid), phloretin, niacinamide, hyaluronic acid, panthenol, and pigment actives (eg, tranexamic acid) may all be used. Because mild disruption of the barrier has occurred, their access to deeper tissues is enhanced. Actives may be more potent in this scenario of barrier disruption; hence, the use of agents would be appropriate for use with broken skin.

Following EBD procedures that cause moderate skin barrier disruption (Figure 3), agents should aim at efficacy but also to promote repair of the skin barrier. The use of antioxidants and peptides may be advantageously applied to increase their efficacy, whereas exosomes, stem-cell extracts, growth factors, and hypoallergenic botanicals (eg, cucumber extract) may also accelerate skin barrier restoration.

Following EBD procedures that cause severe skin barrier disruption (Figure 4), the use of occlusive moisturizers may be necessary to act as a temporary artificial barrier, whereas exosomes, stem-cell extracts, growth factors, and peptides may accelerate the regeneration of the skin barrier, with further research needed to quantify this.

### Skin Aftercare

Skincare requirements in the days to weeks following an EBD treatment are dependent on the type of EBD treatment used and the consequent degree of skin barrier disruption it can cause. Appropriate skincare following EBD procedures can both strengthen skin barrier function and enhance and maintain the results achieved by the EBD procedure.

Following EBD procedures that do not usually cause any skin barrier disruption (Figure 1), a skin-type appropriate skincare routine, which includes a pH-balanced cleanser, moisturizer, and sunscreen, should be maintained. Prescription actives that the patient had been using before treatment can be continued as prescribed.

Following EBD procedures that cause mild skin barrier disruption (Figure 2), a skincare routine comprised of a pH-balanced cleanser, neutral pH moisturizer (lotion or light cream with ceramides and glycerine), sunscreen, antioxidants, Vitamin B3, Vitamin B5, and hyaluronic acid is advised. The use of toners should be avoided because they can cause irritation and prolonged healing. The use of prescription actives should be avoided for 4 to 12 days postprocedure as some medicines, such as retinoids, AHAs, and hydroquinone, have been known to cause irritation and therefore may adversely impact healing.

Following EBD procedures that cause moderate skin barrier disruption (Figure 3), a gentle cleanser and a richer cream-based moisturizer with ceramides, glycerine, petrolatum, and dimethicone can be used. Care should be taken to only use products tested for use on broken skin because there will be a higher risk of skin absorption following these EBD procedures. The use of toners, sodium-lauryl sulfates, and allergenics should be avoided to prevent irritation, increased inflammation, or sensitization. Caution should be taken with the use of irritating and prescription active ingredients, including retinoids, hydroquinone, and acids. These actives should be avoided for 2 to 3 weeks postprocedure in order to avoid any additional inflammation or irritation of the skin during the healing process.

Following EBD procedures that cause severe skin barrier disruption (Figure 4), an occlusive moisturizer containing dimethicone or petrolatum is recommended. Dimethicone is a high-molecular-weight silicone. Because of its high molecular weight and hydrophobicity, dimethicone is poorly absorbed by skin, thus acting as a barrier to prevent water loss and the penetration of exogenous substances.^43^ Dimethicone is widely used for skin repair.^44^ The use of highly occlusive skin products, such as petrolatum, should be used cautiously on acne-prone skin, which may progress to acne breakout. Highly occlusive skin products may also cause milia; hence, they should only be used for up to 5 to 7 days post-EBD procedure. After this, a less occlusive moisturizer should be used. The use of irritating and prescription actives should be avoided for ≥24 days postprocedure or as determined by an experienced practitioner.

## LIMITATIONS

The available evidence for the use of most skincare actives for facial EBD treatment is sparse, and mainly consists of studies with small sample size, making recommendations solely based on clinical evidence difficult. There is a need for more large randomized controlled trials in this area.

The authors recognize that it is difficult to make generalized skincare recommendations because of the wide range of EBD treatments available. This is why recommendations were divided based on the degree of skin barrier disruption. These recommendations are general and may not suit everyone, especially in regions with broad ethnic skin color diversity and skin types, such as Australia. Therefore, clinician experience and discretion are paramount.

The authors of this review are practitioners within Australia only, and their experience extends to this region. As such, recommendations presented should be considered within the context of individual regions.

## CONCLUSIONS

A multimodal approach, which includes integrated skincare, is needed for the optimization of EBD treatment outcomes. The algorithms provided here are aimed to assist clinicians in optimizing integrated skincare recommendations to enhance EBD treatment outcomes, promote healing, and reduce the likelihood of adverse events. Skincare recommendations made to patients should be based on the condition of the patient's skin, as well as the magnitude of skin barrier disruption caused by the EBD procedure. The greater the disruption caused to the skin barrier, the greater care needed in choosing appropriate skincare products with suitable actives to promote barrier repair and avoid further irritation. Prescription actives should be avoided for an appropriate period following EBD procedures; this period is dependent on the EBD treatment type and degree of skin barrier disruption it can cause.
